# 3,4-Bis(2-pyrid­yl)-5-(3-pyrid­yl)-4*H*-1,2,4-triazole

**DOI:** 10.1107/S1600536811014140

**Published:** 2011-04-22

**Authors:** Jing-Min Wu, Wei Guo, Cheng-Peng Li

**Affiliations:** a College of Chemistry, Tianjin Key Laboratory of Structure and Performance for Functional Molecules, Tianjin Normal University, Tianjin 300387, People’s Republic of China

## Abstract

In the title mol­ecule, C_17_H_12_N_6_, the 2-pyridyl rings in the 3- and 4-positions and the 3-pyridyl ring in the 5-position make dihedral angles of 29.78 (16), 67.06 (16) and 32.97 (16)°, respectively, with the triazole group. The dihedral angle between the two 2-pyridyl rings is 65.72 (15)°. The dihedral angles between the 3-pyridyl ring and the two 2-pyridyl rings in the 3- and 4-positions are 61.28 (15) and 63.11 (15)°, respectively. In the crystal, C—H⋯π and π–π inter­actions [centroid-centroid distance = 3.6248 (19) Å] link the mol­ecules, forming a two-dimensional network.

## Related literature

For the synthesis of the title compound, see: Klingele & Brooker (2004 ▶). For related structures and background references, see: Guo *et al.* (2010 ▶); Yang *et al.* (2010 ▶).
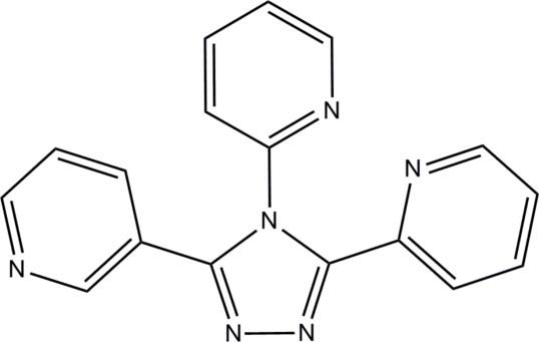


## Experimental

### 

#### Crystal data

C_17_H_12_N_6_
*M* *_r_* = 300.33
Monoclinic, 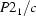

*a* = 5.7621 (9) Å
*b* = 15.250 (3) Å
*c* = 16.640 (3) Å
β = 105.023 (5)°
*V* = 1412.2 (4) Å^3^
*Z* = 4
Mo *K*α radiation
μ = 0.09 mm^−1^
*T* = 296 K
0.28 × 0.22 × 0.20 mm

#### Data collection

Bruker SMART CCD area-detector diffractometer
Absorption correction: multi-scan (*SADABS*; Sheldrick, 1996 ▶) *T*_min_ = 0.975, *T*_max_ = 0.982
6865 measured reflections
2496 independent reflections
1407 reflections with *I* > 2σ(*I*)
*R*_int_ = 0.046

#### Refinement

*R*[*F* ^2^ > 2σ(*F* ^2^)] = 0.050
*wR*(*F* ^2^) = 0.129
*S* = 1.08
2496 reflections
209 parameters
H-atom parameters constrained
Δρ_max_ = 0.15 e Å^−3^
Δρ_min_ = −0.16 e Å^−3^

### 

Data collection: *SMART* (Bruker, 2007 ▶); cell refinement: *SAINT* (Bruker, 2007 ▶); data reduction: *SAINT*; program(s) used to solve structure: *SHELXS97* (Sheldrick, 2008 ▶); program(s) used to refine structure: *SHELXL97* (Sheldrick, 2008 ▶); molecular graphics: *DIAMOND* (Brandenburg, 1999 ▶); software used to prepare material for publication: *SHELXTL* (Sheldrick, 2008 ▶).

## Supplementary Material

Crystal structure: contains datablocks I, global. DOI: 10.1107/S1600536811014140/su2269sup1.cif

Structure factors: contains datablocks I. DOI: 10.1107/S1600536811014140/su2269Isup2.hkl

Additional supplementary materials:  crystallographic information; 3D view; checkCIF report

## Figures and Tables

**Table 1:** Hydrogen-bond geometry (Å, °) *Cg*2 and *Cg*4 are the centroids of the N1/C8–C12 and N6/C13–C17 rings, respectively.

*D*—H⋯*A*	*D*—H	H⋯*A*	*D*⋯*A*	*D*—H⋯*A*
C3—H3⋯*Cg*2^i^	0.93	2.94	3.765 (4)	149
C4—H4⋯*Cg*4	0.93	2.92	3.616 (3)	133

Symmetry code: (i) 
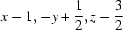
.

**Fig. 1.**
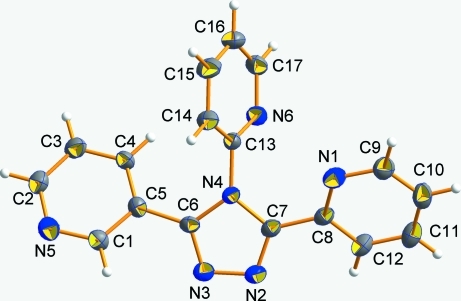
Molecular structure of the title molecule showing the numbering scheme and displacement ellipsoids drawn at the 30% probability level.

**Fig. 2.**
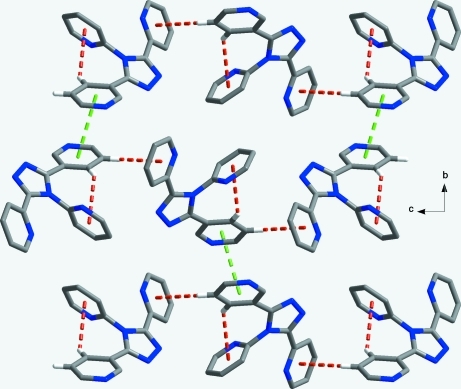
A view along the a axis of the C—H···π (red dashed lines) and π–π (green dashed lines) interactions in the crystal packing of the title compound (see Table 1 for details).

## supplementary crystallographic information

### Comment

In continuation of our work on tripyridine-substituted triazole derivatives (Guo *et al.*, 2010; Yang *et al.*, 2010), we now describe the synthesis and crystal structure of the title compound. It consists of two 2-pyridyl groups and one 3-pyridyl ring attached to a triazole ring (Fig. 1).

The three pyridyl rings in the 3-, 4-, and 5-positions deviate from the triazole ring by 29.78 (16)°, 67.06 (16)°, and 32.97 (16)°, respectively. The dihedral angle between the two 2-pyridyl groups is 65.72 (15)°. In addition, the dihedral angles between the 3-pyridyl ring and the two 2-pyridyl rings in the 3- and 4-positions are 61.28 (15)° and 63.11 (15)°, respectively.

In the crystal, there exists a π–π interaction involving the 2-pyridyl rings in 3-positions of molecules related by an inversion center [centroid-centroid distance = 3.6248 (19) Å]. The molecular packing is also stabilized by two types of C—H···π interactions; the intramolecular C4—H4···*Cg*4 [*Cg*4 is the centroid of pyridine ring (N6/C13-C17)] and the intermolecular C3—H3···*Cg*2^i^ [*Cg*2 is the centroid of pyridine ring (N1/C8-C12)] interactions (see Table 1 and Fig. 2 for details). This leads to the formation of a two-dimensional network.

### Experimental

The title compound was prepared from a mixture of *N*-(pyridin-2-yl)pyridine-2-carbothioamide and pyridine-3-carbohydrazide using the method described by Klingele *et al.* (2004).

### Refinement

All H atoms were initially located in a difference Fourier map. The C—H atoms were then constrained to ideal geometry and refined as riding atoms: C—H = 0.93 Å, with *U*_iso_(H) = 1.2*U*eq(C).

### Crystal data

**Table:**

C_17_H_12_N_6_	*F*(000) = 624
*M**_r_* = 300.33	*D*_x_ = 1.413 Mg m^−^^3^
Monoclinic, *P*2_1_/*c*	Mo *K*α radiation, λ = 0.71073 Å
Hall symbol: -P 2ybc	Cell parameters from 1374 reflections
*a* = 5.7621 (9) Å	θ = 2.5–22.0°
*b* = 15.250 (3) Å	µ = 0.09 mm^−^^1^
*c* = 16.640 (3) Å	*T* = 296 K
β = 105.023 (5)°	Block, colourless
*V* = 1412.2 (4) Å^3^	0.28 × 0.22 × 0.20 mm
*Z* = 4	

### Data collection

**Table:**

Bruker SMART CCD area-detector diffractometer	2496 independent reflections
Radiation source: fine-focus sealed tube	1407 reflections with *I* > 2σ(*I*)
graphite	*R*_int_ = 0.046
φ and ω scans	θ_max_ = 25.0°, θ_min_ = 1.8°
Absorption correction: multi-scan (*SADABS*; Sheldrick, 1996)	*h* = −5→6
*T*_min_ = 0.975, *T*_max_ = 0.982	*k* = −18→18
6865 measured reflections	*l* = −19→17

### Refinement

**Table:**

Refinement on *F*^2^	Secondary atom site location: difference Fourier map
Least-squares matrix: full	Hydrogen site location: inferred from neighbouring sites
*R*[*F*^2^ > 2σ(*F*^2^)] = 0.050	H-atom parameters constrained
*wR*(*F*^2^) = 0.129	*w* = 1/[σ^2^(*F*_o_^2^) + (0.0385*P*)^2^ + 0.6358*P*] where *P* = (*F*_o_^2^ + 2*F*_c_^2^)/3
*S* = 1.08	(Δ/σ)_max_ < 0.001
2496 reflections	Δρ_max_ = 0.15 e Å^−^^3^
209 parameters	Δρ_min_ = −0.16 e Å^−^^3^
0 restraints	Extinction correction: *SHELXL97* (Sheldrick, 2008), Fc^*^=kFc[1+0.001xFc^2^λ^3^/sin(2θ)]^-1/4^
Primary atom site location: structure-invariant direct methods	Extinction coefficient: 0.0124 (17)

### Special details

**Table:**

Geometry. All e.s.d.'s (except the e.s.d. in the dihedral angle between two l.s. planes) are estimated using the full covariance matrix. The cell e.s.d.'s are taken into account individually in the estimation of e.s.d.'s in distances, angles and torsion angles; correlations between e.s.d.'s in cell parameters are only used when they are defined by crystal symmetry. An approximate (isotropic) treatment of cell e.s.d.'s is used for estimating e.s.d.'s involving l.s. planes.
Refinement. Refinement of *F*^2^ against ALL reflections. The weighted *R*-factor *wR* and goodness of fit *S* are based on *F*^2^, conventional *R*-factors *R* are based on *F*, with *F* set to zero for negative *F*^2^. The threshold expression of *F*^2^ > σ(*F*^2^) is used only for calculating *R*-factors(gt) *etc*. and is not relevant to the choice of reflections for refinement. *R*-factors based on *F*^2^ are statistically about twice as large as those based on *F*, and *R*- factors based on ALL data will be even larger.

### Fractional atomic coordinates and isotropic or equivalent isotropic displacement parameters (Å^2^)

**Table:**

	*x*	*y*	*z*	*U*_iso_*/*U*_eq_	
N1	0.8676 (5)	0.8964 (2)	0.69324 (16)	0.0620 (8)	
N2	0.6880 (5)	0.68510 (18)	0.75149 (15)	0.0531 (7)	
N3	0.4816 (5)	0.63917 (17)	0.71292 (16)	0.0525 (7)	
N4	0.5220 (4)	0.74876 (16)	0.63138 (13)	0.0402 (6)	
N5	−0.2135 (5)	0.57144 (18)	0.56487 (19)	0.0646 (8)	
N6	0.6492 (4)	0.79687 (17)	0.51665 (15)	0.0527 (7)	
C1	−0.0072 (6)	0.6053 (2)	0.6135 (2)	0.0524 (8)	
H1	0.0195	0.6012	0.6710	0.063*	
C2	−0.2431 (6)	0.5784 (2)	0.4816 (2)	0.0572 (9)	
H2	−0.3822	0.5557	0.4463	0.069*	
C3	−0.0776 (6)	0.6174 (2)	0.4471 (2)	0.0585 (9)	
H3	−0.1055	0.6206	0.3896	0.070*	
C4	0.1285 (5)	0.6514 (2)	0.49739 (19)	0.0479 (8)	
H4	0.2420	0.6781	0.4746	0.057*	
C5	0.1657 (5)	0.64570 (19)	0.58214 (19)	0.0436 (8)	
C6	0.3855 (5)	0.6773 (2)	0.64142 (17)	0.0418 (7)	
C7	0.7095 (5)	0.7501 (2)	0.70183 (18)	0.0438 (8)	
C8	0.9078 (5)	0.8133 (2)	0.72220 (17)	0.0449 (8)	
C9	1.0537 (7)	0.9537 (2)	0.7146 (2)	0.0653 (10)	
H9	1.0303	1.0110	0.6951	0.078*	
C10	1.2745 (6)	0.9304 (3)	0.7638 (2)	0.0680 (10)	
H10	1.3979	0.9715	0.7771	0.082*	
C11	1.3130 (6)	0.8464 (3)	0.7933 (2)	0.0639 (10)	
H11	1.4620	0.8297	0.8268	0.077*	
C12	1.1283 (5)	0.7875 (2)	0.77272 (19)	0.0547 (9)	
H12	1.1503	0.7303	0.7925	0.066*	
C13	0.4854 (5)	0.80537 (19)	0.55974 (17)	0.0395 (7)	
C14	0.2895 (6)	0.8593 (2)	0.5398 (2)	0.0525 (9)	
H14	0.1811	0.8621	0.5726	0.063*	
C15	0.2599 (7)	0.9094 (2)	0.4689 (2)	0.0685 (11)	
H15	0.1300	0.9475	0.4529	0.082*	
C16	0.4233 (7)	0.9027 (2)	0.4218 (2)	0.0689 (11)	
H16	0.4053	0.9358	0.3737	0.083*	
C17	0.6135 (6)	0.8462 (2)	0.4475 (2)	0.0625 (10)	
H17	0.7234	0.8417	0.4154	0.075*	

### Atomic displacement parameters (Å^2^)

**Table:**

	*U*^11^	*U*^22^	*U*^33^	*U*^12^	*U*^13^	*U*^23^
N1	0.0686 (19)	0.064 (2)	0.0505 (18)	0.0029 (17)	0.0101 (14)	−0.0032 (15)
N2	0.0565 (17)	0.0589 (18)	0.0425 (15)	0.0064 (15)	0.0106 (13)	0.0061 (14)
N3	0.0548 (17)	0.0546 (18)	0.0482 (17)	0.0043 (14)	0.0133 (14)	0.0059 (14)
N4	0.0393 (14)	0.0462 (15)	0.0344 (14)	0.0038 (12)	0.0082 (11)	0.0007 (12)
N5	0.0652 (19)	0.0567 (19)	0.072 (2)	−0.0041 (15)	0.0186 (17)	−0.0024 (16)
N6	0.0512 (16)	0.0626 (19)	0.0468 (16)	0.0017 (13)	0.0174 (13)	0.0008 (14)
C1	0.059 (2)	0.049 (2)	0.053 (2)	−0.0062 (17)	0.0216 (18)	−0.0058 (16)
C2	0.049 (2)	0.058 (2)	0.060 (2)	0.0030 (17)	0.0076 (18)	−0.0059 (18)
C3	0.054 (2)	0.073 (3)	0.049 (2)	0.0043 (18)	0.0135 (18)	0.0028 (17)
C4	0.0454 (19)	0.054 (2)	0.0474 (19)	−0.0025 (16)	0.0183 (16)	0.0041 (15)
C5	0.0412 (18)	0.0410 (19)	0.0516 (19)	0.0034 (15)	0.0175 (16)	−0.0033 (15)
C6	0.0460 (18)	0.0450 (19)	0.0377 (17)	0.0049 (16)	0.0166 (14)	0.0034 (15)
C7	0.0448 (18)	0.0488 (19)	0.0374 (17)	0.0069 (16)	0.0103 (14)	−0.0016 (16)
C8	0.0463 (18)	0.051 (2)	0.0362 (17)	0.0074 (16)	0.0092 (14)	−0.0033 (15)
C9	0.074 (3)	0.060 (2)	0.057 (2)	−0.011 (2)	0.008 (2)	−0.0068 (18)
C10	0.061 (2)	0.079 (3)	0.064 (2)	−0.018 (2)	0.016 (2)	−0.024 (2)
C11	0.050 (2)	0.074 (3)	0.062 (2)	0.003 (2)	0.0043 (18)	−0.019 (2)
C12	0.052 (2)	0.058 (2)	0.050 (2)	0.0095 (18)	0.0043 (16)	−0.0075 (16)
C13	0.0370 (16)	0.0458 (19)	0.0362 (16)	−0.0002 (15)	0.0105 (14)	−0.0012 (14)
C14	0.0479 (19)	0.059 (2)	0.053 (2)	0.0133 (17)	0.0176 (16)	0.0079 (17)
C15	0.065 (2)	0.068 (3)	0.067 (2)	0.019 (2)	0.008 (2)	0.020 (2)
C16	0.072 (3)	0.081 (3)	0.050 (2)	−0.005 (2)	0.010 (2)	0.022 (2)
C17	0.065 (2)	0.081 (3)	0.047 (2)	−0.014 (2)	0.0237 (18)	0.0002 (19)

### Geometric parameters (Å, °)

**Table:**

N1—C8	1.354 (4)	C4—H4	0.9300
N1—C9	1.357 (4)	C5—C6	1.470 (4)
N2—C7	1.316 (4)	C7—C8	1.467 (4)
N2—N3	1.386 (3)	C8—C12	1.386 (4)
N3—C6	1.311 (3)	C9—C10	1.369 (5)
N4—C7	1.373 (3)	C9—H9	0.9300
N4—C6	1.379 (4)	C10—C11	1.371 (5)
N4—C13	1.442 (3)	C10—H10	0.9300
N5—C2	1.355 (4)	C11—C12	1.366 (4)
N5—C1	1.355 (4)	C11—H11	0.9300
N6—C13	1.331 (3)	C12—H12	0.9300
N6—C17	1.345 (4)	C13—C14	1.367 (4)
C1—C5	1.384 (4)	C14—C15	1.378 (4)
C1—H1	0.9300	C14—H14	0.9300
C2—C3	1.370 (4)	C15—C16	1.376 (5)
C2—H2	0.9300	C15—H15	0.9300
C3—C4	1.366 (4)	C16—C17	1.373 (5)
C3—H3	0.9300	C16—H16	0.9300
C4—C5	1.373 (4)	C17—H17	0.9300
			
C8—N1—C9	117.3 (3)	N1—C8—C7	118.8 (3)
C7—N2—N3	107.4 (2)	C12—C8—C7	119.3 (3)
C6—N3—N2	107.8 (2)	N1—C9—C10	122.6 (3)
C7—N4—C6	104.8 (2)	N1—C9—H9	118.7
C7—N4—C13	127.7 (2)	C10—C9—H9	118.7
C6—N4—C13	127.4 (2)	C9—C10—C11	119.7 (3)
C2—N5—C1	116.1 (3)	C9—C10—H10	120.2
C13—N6—C17	115.7 (3)	C11—C10—H10	120.2
N5—C1—C5	123.3 (3)	C12—C11—C10	118.8 (3)
N5—C1—H1	118.3	C12—C11—H11	120.6
C5—C1—H1	118.3	C10—C11—H11	120.6
N5—C2—C3	123.1 (3)	C11—C12—C8	119.8 (3)
N5—C2—H2	118.5	C11—C12—H12	120.1
C3—C2—H2	118.5	C8—C12—H12	120.1
C4—C3—C2	119.8 (3)	N6—C13—C14	125.8 (3)
C4—C3—H3	120.1	N6—C13—N4	114.5 (2)
C2—C3—H3	120.1	C14—C13—N4	119.7 (3)
C3—C4—C5	118.9 (3)	C13—C14—C15	116.9 (3)
C3—C4—H4	120.5	C13—C14—H14	121.6
C5—C4—H4	120.5	C15—C14—H14	121.6
C4—C5—C1	118.7 (3)	C16—C15—C14	119.7 (3)
C4—C5—C6	123.1 (3)	C16—C15—H15	120.1
C1—C5—C6	118.2 (3)	C14—C15—H15	120.1
N3—C6—N4	109.9 (3)	C17—C16—C15	118.6 (3)
N3—C6—C5	123.5 (3)	C17—C16—H16	120.7
N4—C6—C5	126.6 (3)	C15—C16—H16	120.7
N2—C7—N4	110.1 (3)	N6—C17—C16	123.4 (3)
N2—C7—C8	123.0 (3)	N6—C17—H17	118.3
N4—C7—C8	126.9 (3)	C16—C17—H17	118.3
N1—C8—C12	121.8 (3)		
			
C7—N2—N3—C6	0.2 (3)	C9—N1—C8—C12	1.0 (4)
C2—N5—C1—C5	0.5 (5)	C9—N1—C8—C7	178.7 (3)
C1—N5—C2—C3	−0.3 (5)	N2—C7—C8—N1	−149.0 (3)
N5—C2—C3—C4	0.0 (5)	N4—C7—C8—N1	30.4 (4)
C2—C3—C4—C5	0.1 (5)	N2—C7—C8—C12	28.8 (4)
C3—C4—C5—C1	0.2 (4)	N4—C7—C8—C12	−151.7 (3)
C3—C4—C5—C6	177.6 (3)	C8—N1—C9—C10	−0.4 (5)
N5—C1—C5—C4	−0.5 (5)	N1—C9—C10—C11	−0.1 (5)
N5—C1—C5—C6	−178.0 (3)	C9—C10—C11—C12	0.0 (5)
N2—N3—C6—N4	−0.7 (3)	C10—C11—C12—C8	0.5 (5)
N2—N3—C6—C5	178.8 (3)	N1—C8—C12—C11	−1.1 (5)
C7—N4—C6—N3	0.8 (3)	C7—C8—C12—C11	−178.8 (3)
C13—N4—C6—N3	176.5 (3)	C17—N6—C13—C14	−0.3 (4)
C7—N4—C6—C5	−178.6 (3)	C17—N6—C13—N4	177.2 (3)
C13—N4—C6—C5	−3.0 (4)	C7—N4—C13—N6	65.1 (4)
C4—C5—C6—N3	−145.5 (3)	C6—N4—C13—N6	−109.6 (3)
C1—C5—C6—N3	31.9 (4)	C7—N4—C13—C14	−117.1 (3)
C4—C5—C6—N4	33.9 (5)	C6—N4—C13—C14	68.2 (4)
C1—C5—C6—N4	−148.7 (3)	N6—C13—C14—C15	−0.2 (5)
N3—N2—C7—N4	0.3 (3)	N4—C13—C14—C15	−177.6 (3)
N3—N2—C7—C8	179.8 (3)	C13—C14—C15—C16	0.5 (5)
C6—N4—C7—N2	−0.7 (3)	C14—C15—C16—C17	−0.4 (5)
C13—N4—C7—N2	−176.3 (3)	C13—N6—C17—C16	0.5 (5)
C6—N4—C7—C8	179.8 (3)	C15—C16—C17—N6	−0.2 (5)
C13—N4—C7—C8	4.2 (5)		

### Hydrogen-bond geometry (Å, °)

**Table:**

Cg2 and Cg4 are the centroids of the N1/C8–C12 and N6/C13–C17 rings, respectively.

**Table:**

*D*—H···*A*	*D*—H	H···*A*	*D*···*A*	*D*—H···*A*
C3—H3···Cg2^i^	0.93	2.94	3.765 (4)	149
C4—H4···Cg4	0.93	2.92	3.616 (3)	133

Symmetry codes: (i) *x*−1, −*y*+1/2, *z*−3/2.
